# Supplementary material for: Artificial sediments enable reproducible cultivation and recapitulate ecological interactions of cable bacteria
Source: ISME Commun. 2026 Jul 8;6(1):ycag199. doi: 10.1093/ismeco/ycag199 (PMC13477219; doi:10.1093/ismeco/ycag199)
Supplement: Supplementary file 1 [file supplementary_material_ycag199.zip › 260624_SupplementalInformation.docx]

**Supplemental Information**

**Figure S1.** Vertical chemical profiles of non-inoculated artificial sediments. Oxygen (black), sulfide (red) and pH (blue) profiles were measured for artificial sediments for **(A)** AS1 and **(B)** AS2 of non-inoculated sediments immediately before inoculation (dotted line) and following 14 days of incubation (dashed-dot line, same data as reported in Figure 1 for reference).

**Figure S2.** Vertical chemical profiles of artificial sediments for enrichments 4-6. Oxygen (black), sulfide (red) and pH (blue) profiles were measured for artificial sediments for AS2 of freshwater *E. aureum* for quarternary (solid line), quinary (dash-dotted line), senary (dashed line) enrichments.

**Figure S3.** Species richness rarefaction curves. The number of observed ASVs varies as read depth increases for the natural sediment (black) and artificial sediments AS1 (red) and AS2 (blue).

**Figure S4.** Alpha-diversity metrics (Observed ASVs, Shannon Index, and Simpson Index) for the freshwater enrichments on natural sediment (black) and artificial sediments AS1 (red) and AS2 (blue).

**Figure S5.** Variation in the microbial community across artificial sediment enrichments. **(A)** robust Aitchison distances^39^ between each artificial sediment enrichment relative to the natural sediment community **(B)** robust principal coordinate analysis (PC1 and PC2) among sediment enrichments. AS1 are blue, AS2 are red, NS is black. enrichment 1 are circles, enrichment 2 are squares, enrichment 3 are triangles. Bar plots and error bars represent the mean and standard deviation, respectively, and individual replicates shown as circles.

**Figure S6.** Spearman correlation of taxa abundance rank between serial enrichments for **(A)** AS1 and **(B)** AS2**.** Colored points and error bars represent the mean and standard deviation, respectively, of Spearman rho for all possible pairs of replicates between serial enrichments with individual replicate pairs shown as black circles. Spearman correlations were calculated using centered log-ratio transformed read counts using a pseudocount of 0.5 added to all zero counts.

**Figure S7.** Heat map depicting the log abundance of the top 25 taxa within the natural sediment. Top bar plot depicts the fraction of total reads from each sample displayed in each column of the heatmap. Data are relative abundances from individual cores (n=1 the natural sediment inoculum).

**Figure S8.** Heat map depicting the log abundance of putative flocking genera identified in Bjerg et al.^38^ and Lustermans et al.^41^ found in natural sediments as well as AS1 and AS2 that were not inoculated with *E. aureum* (C) or the primary (E1), secondary (E2), or tertiary (E3) serial enrichment of artificial sediments inoculated with *E. aureum*. Data are relative abundances from individual replicate cores (n=3 for all serial enrichments except AS2-E2 which has n=2, n=1 for negative controls and the natural sediment inoculum).

**Figure S9.** Phylogenetic tree of cable bacterial 16S rRNA genes. Phylogeny was constructed by aligning 16S rRNA gene sequences (>1200 bp) of cable bacteria and related taxa (n=107) from Ley et al.^32^ as well as the recently identified *Ca*. Electrothrix yaqonensis YB6^33^ and the *Ca*. Electrothrix sp. NJ1 recovered in this study using MUSCLE^31^. A maximum likelihood tree was constructed with IQ-TREE (v1.6.12)^34^, employing the TIM3e+I+G4 model and 1,000 ultrafast bootstrap replicates. Tree was visualized using iTOL^35^. The two cable bacteria used in this study *Electronema aureum* GS and *Ca.* Electrothrix sp. NJ1 are colored in red. Scale bar represents 10% estimated sequence divergence.

**Figure S10.** Pairwise sequence identity matrix of cable bacteria 16s rRNA genes. Clustering is based on the phylogenetic tree in Figure S5. The two cable bacteria used in this study *Electronema aureum* and *Ca.* Electrothrix sp. NJ1 are colored in red.

**Figure S11.** Scanning electron micrographs of *Ca.* Electrothrix sp. NJ1 enriched in natural sediments. SEM imaging was performed using a Quanta 200 FEG Environmental SEM (Thermo Fisher Scientific; formerly FEI). Cable bacterium filaments were carefully isolated from sediment columns using glass hooks as previously described^14^ and sequentially rinsed through six droplets of Milli-Q water to remove adhering sediment particles. The cleaned filaments were transferred onto 0.4 μm polycarbonate track-etched membrane filters (Whatman™ Nuclepore™, Cat. No. 10417106) and dehydrated through a graded ethanol series (10%, 30%, 50%, 70%, 90%, and 100%), followed by air drying overnight. SEM images were acquired at an accelerating voltage of 5 kV, a working distance of 10.5 mm, and a spot size of 3.

**Video S1.** Live cell phase contrast microscopy of single celled organisms flocking around an *E. aureum* filament grown in AS2 in a trench slide device. Video is at 8x speed.

| **Component** | **Concentration (g/L)** | **Concentration** |
| --- | --- | --- |
| CaSO4 * 2H2O | 0.12 | 696 µM |
| EDTA | 0.01 | 34.2 µM |
| MgSO4 * 7H2O | 0.2 | 811.45 µM |
| NaCl | 0.016 | 273.8 µM |
| CaCl2 * 2H2O | 0.264 | 1.8 mM |
| Na2HPO4 | 0.071 | 0.5 mM |
| KH2PO4 | 0.068 | 0.5 mM |
| NaHCO3 | 0.168 | 2 mM |
| CH3COONa | 0.004 | 50 µM |
| NH4Cl | 0.003 | 50 µM |
| NaNO3 | 0.004 | 50 µM |
| FeCl3 solution (0.29 g/L) | 2 mL | 1.78 µM |
| Micronutrient solution | - | 1x |
| Vitamin solution | - | 1x |
| Deionized water | 1000 mL |  |

**Table S1.** Freshwater benthic medium (FWBM) recipe.

| **Component** | **Concentration (mL/L or g/L)** |
| --- | --- |
| H2SO4, sulfuric acid (>98%) | 0.5 mL |
| MnSO4 * H2O | 2.2 g |
| ZnSO4 * 7 H2O | 0.5 g |
| H3BO3 | 0.5 g |
| CuSO4 * 5 H2O | 0.025 g |
| Na2MoO4 * 2 H2O | 0.025 g |
| CoCl2 * 6 H2O | 0.045 g |
| NiCl2* 6 H2O | 0.025 g |
| deionized water | 1000 mL |

**Table S2.** Micronutrient solution recipe. The 1000x micronutrient solution consisted of the following (per 1000 mL): sulfuric acid (0.5 mL), manganese sulfate monohydrate (2.2 g), zinc sulfate heptahydrate (0.5 g), boric acid (0.5 g), cupric sulfate pentahydrate (0.025 g), sodium molybdate dihydrate (0.025 g), cobalt chloride hexahydrate (0.045 g), nickel chloride hexahydrate (0.025 g). The solution was filter sterilized using a 0.2 µm PES filter and stored at 4°C until use.

| **Component** | **Volume (mL)** |
| --- | --- |
| 0.1 mg ml^-1^ B12 | 1 mL |
| 0.1 mg ml^-1^ inositol | 1 mL |
| 0.1 mg ml^-1^ biotin | 1 mL |
| 0.1 mg ml^-1^ folic acid | 1 mL |
| 1 mg ml^-1^ p-aminobenzoic acid | 1 mL |
| 10 mg ml^-1^ nicotinic acid | 1 mL |
| 10 mg ml^-1^ d-pantothenate | 1 mL |
| 20 mg ml^-1^ thiamine | 1 mL |
| deionized water | 92 mL |

**Table S3.** Vitamin solution recipe. The 2400x vitamin solution consisted of individual stock solutions of the following (per 100 mL): 0.1 mg ml^-1^ B12, 0.1 mg ml^-1^ inositol, 0.1 mg ml^-1^ biotin, 0.1 mg ml^-1^ folic acid, 1 mg ml^-1^ p-aminobenzoic acid, 10 mg ml^-1^ nicotinic acid, 10 mg ml^-1^ d-pantothenate, 20 mg ml^-1^ thiamine. Stock solutions of individual components were initially prepared in deionized water and stored at -80°C until use. To obtain the final stock solution, 1 ml each of the above stock solutions were added to 92 ml deionized water, followed by filter sterilization using a 0.2 µm PES filter. The solution was stored at 4°C until use.

**Table S4.** Table of ASVs observed across all samples as well as ASVs that have been grouped at the genus level.

**Table S5.** Table of ASVs that are commonly observed in this study as well as Lustermans et al.^41^

**Table S6.** Table of Spearman’s correlation coefficients for all ASVs relative to *E. aureum* (ASV_15) across all samples from this study. Spearman correlations were calculated using centered log-ratio transformed read counts using a pseudocount of 0.5 added to all zero counts.
